# Inflammatory arthritis complicating galactosialidosis: a case report

**DOI:** 10.1186/s41927-021-00208-0

**Published:** 2021-10-11

**Authors:** F. Verkuil, A. M. Bosch, P. A. A. Struijs, R. Hemke, J. M. van den Berg

**Affiliations:** 1 Emma Children’s Hospital, Amsterdam University Medical Centers, location Academic Medical Center, Pediatric Immunology, Rheumatology and Infectious Diseases, University of Amsterdam, Meibergdreef 9, 1105 AZ Amsterdam, The Netherlands; 2 Radiology and Nuclear Medicine, Amsterdam University Medical Centers, location Academic Medical Center, Amsterdam Movement Sciences, University of Amsterdam, Meibergdreef 9, 1105 AZ Amsterdam, The Netherlands; 3 Emma Children’s Hospital, Amsterdam University Medical Centers, location Academic Medical Center, Pediatric Metabolic Diseases, University of Amsterdam, Meibergdreef 9, 1105 AZ Amsterdam, The Netherlands; 4 Orthopedic Surgery, Amsterdam University Medical Centers, location Academic Medical Center, University of Amsterdam, Meibergdreef 9, 1105 AZ Amsterdam, The Netherlands

**Keywords:** Lysosomal storage disorder, Galactosialidosis, Inherited metabolic disorder, Inflammatory arthritis, Joint inflammation, Synovitis, Children

## Abstract

**Background:**

Galactosialidosis (GS) is a rare inherited lysosomal storage disorder (LSD) which is characterized by a defect in the lysosomal glycoprotein catabolism. We report, for the first time, the case of a child affected by GS presenting with recurrent episodes of extensive joint inflammation in both knee joints. The aim of this case-report is to describe the clinical presentation as well as the laboratory, radiologic and microscopic features of this unique presentation of GS. Furthermore, we explore inflammatory mechanisms potentially responsible for the origination of the arthritic joint pathology observed in our patient.

**Case presentation:**

We describe the rare case of a 12-year-old boy diagnosed with GS (late infantile form) who presented with multiple episodes of inflammatory arthritis involving both knees; no other joints were suspected for joint inflammation. Laboratory results did not indicate an autoimmune disorder. Synovial fluid tested negative for any bacterial infection and ruled out a malignancy and crystal-induced arthritis. Microscopic examination of the synovial tissue revealed numerous foamy macrophages with extensive vacuolization, consistent with the previous diagnosis of GS. Treatment consisted of aspiration of excessive joint fluid and subsequent intra-articular injection of triamcinolonhexacetonide with excellent but transient result. Given the evidence of storage products within macrophages of the inflamed synovial tissue and the absence of other etiological clues, GS itself was considered as the primary cause for the relapsing inflammatory joint pathology. According to the restricted data on articular manifestations in GS, to date, GS cannot be linked directly to joint inflammation. Nevertheless, in several other LSDs, the accumulation of storage material has been associated with numerous osteoimmunological changes that might play a role in the pathophysiology of arthritic processes.

**Conclusions:**

We hypothesize that the articular build-up of GS storage products triggered systemic as well as local inflammatory processes, resulting in the extensive inflammatory joint pathology as observed in our patient. Future identification of other patients with GS is required to corroborate the existence of an arthritic clinical phenotype of GS and to assess the underlying pathophysiology.

## Background

Galactosialidosis (GS; MIM #256540) is a rare autosomal recessive lysosomal storage disorder (LSD) characterized by malfunction of the lysosomal glycoprotein degradation and subsequent intra-lysosomal accumulation of sialyloligosaccharides and glycopeptides. In GS, the primary biochemical defect is the deficiency of the lysosomal protective protein/cathepsin A (PPCA), encoded by the *CTSA* gene which is located on chromosome 20q13.1. One of the major functions of PPCA is the formation of a multienzyme complex with alpha-N-acetyl neuraminidase 1 (NEU1; **EC** 3.2.1.18) and beta-galactosidase 1 (GLB1; **EC** 3.2.1.23), which ensures the stability of these enzymes and protects both glycosidases from premature proteolytic post-translational modification [1–3]. Based on the age of onset and clinical features patients are categorized in a distinct GS subtype: the early infantile, the late infantile or the juvenile/adult type. Most patients with GS share some typical clinical features, such as coarse facial features, vertebral deformities, hepatosplenomegaly, cardiac pathology, hearing loss and macular cherry-red-spots [1, 4]. Some studies also describe patients with articular abnormalities [5–8]. Owing to the small number of case reports and brief descriptions of the patients’ phenotype, substantial data about joint pathology in patients with GS is sparse.

Here we report, for the first time, a case of a patient affected by GS who presented with severe osteoarticular manifestations as well as recurrent episodes of inflammatory arthritis in both knees. The aim of this case-report is to describe the clinical presentation as well as the laboratory, radiologic and microscopic features of this rare GS phenotype. Furthermore, we explore inflammatory mechanisms that might play a role in the pathophysiology of the arthritic joint pathology in our patient.

## Case presentation

We report a unique case of a 12-year-old boy, previously diagnosed with GS, who visited the Department of Pediatric Rheumatology with relapsing episodes of progressive swelling in the patellar region in both knees.

**Fig. 1:**
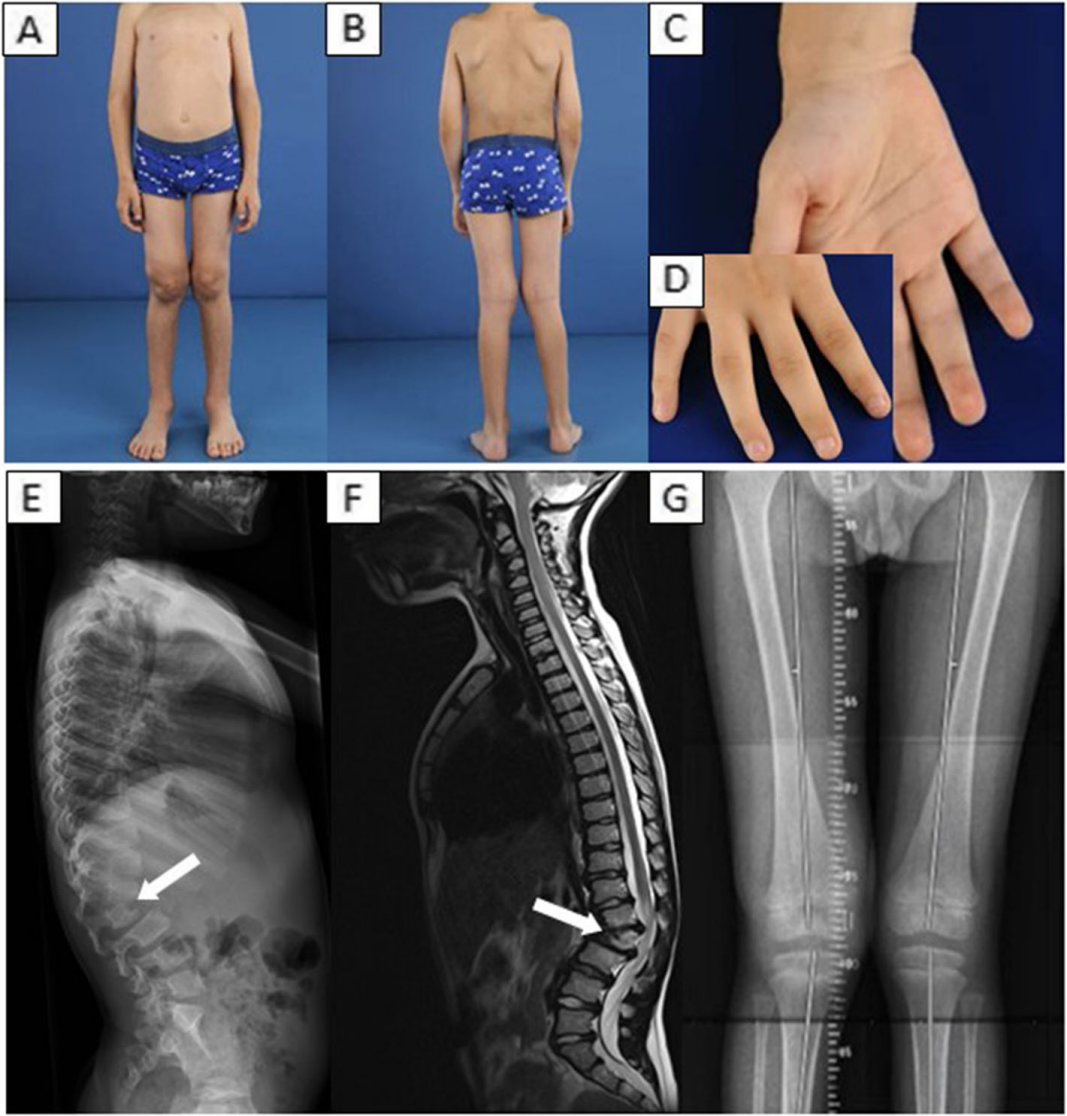
Clinical photographs (age: 8-years-old) revealing genu valgum, hydrops located in the right knee joint (**A** and **B**), ulnar deviation (**C**) and mild contractures of the 2nd, 3rd and 4th proximal interphalangeal joints (**D**). X-rays and MRI showing thoracolumbar kyphosis based on L2 hypoplasia (**E** and **F**) and valgus malalignment in both knees (**G**)

The patient was the first child of healthy, consanguineous, parents with a Turkish ethnic background. Pregnancy and delivery were uneventful and no abnormalities were observed in the first months of life (in particular normal psychomotor development). At 17 months of age, during an admission for an upper airway infection, an enlargement of the liver, a vertebral deformity and coarsening of the facial features were noted; hence an inborn error of metabolism was suspected. Urine oligosaccharides demonstrated elevations of urinary sialyloligosaccharides and subsequent fibroblast enzyme studies revealed deficient activities of PPCA, GLB1 and NEU1, thus confirming the diagnosis of GS. Since there were no prenatal and perinatal abnormalities the patient was classified as having the late infantile form of GS. During the following years the patient developed more typical GS-related clinical characteristics. An overview of the clinical features of GS in our patient is displayed in Table 1. For radiographic images and medical photographs of the musculoskeletal deformities, see Fig. 1.

**Table 1:** Overview of clinical manifestations of GS (late infantile form) in a 12-year-old patient

**Musculoskeletal involvement**	
Coarse facial features	
Short stature	
Vertebral deformity	
*- lumbar kyphosis based on L2 hypoplasia*	
Joint dysplasia	
*- hips, shoulders, knees and wrists*	
Contractures	
*- knees, elbows, wrists and the 2nd, 3rd and 4th proximal interphalangeal joints*	
**Visceral involvement**	
Cardiac pathology	
*- mitral valve regurgitation (mild) and aortic valve regurgitation (mild)*	
**Eye involvement**	
Macular cherry-red-spots

**Fig. 2:**
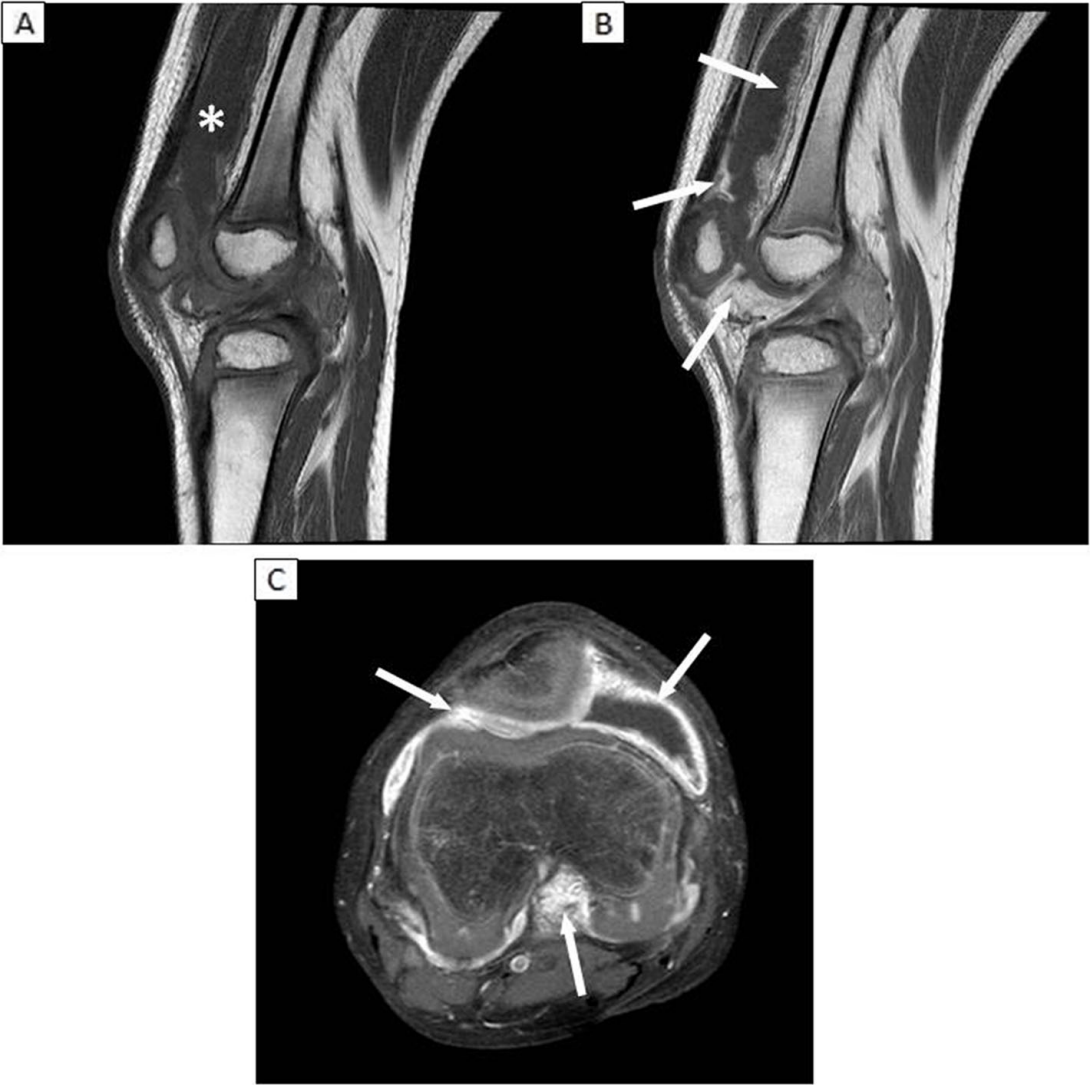
MR images of the right knee joint (obtained at age 8) revealing excessive joint fluid in the suprapatellar recess (see asterisk) and thickened enhancing synovial membrane throughout the entire joint (arrows). **A** Sagittal T1-weighted pre-contrast image; **B** Sagittal T1-weighted post-contrast image showing the thickened and enhancing synovial membrane in the suprapatellar, retropatellar and infrapatellar regions (arrows); **C** Axial T1-weighted fat-suppressed post-contrast image showing thickened enhancing synovial membrane in the retropatellar region and around the cruciate ligaments (arrows)

At the age of 8 years, the boy presented at the Department of Orthopedic Surgery for follow-up in the management of disease-related skeletal and joint pathology (see Table 1), and mild swelling in the right knee was reported for the first time. The initial work-up consisted of whole-leg X-rays, since a disease-related orthopedic cause was suspected. The X-rays showed progression of pre-existing valgus malalignment in both knees and a temporary hemi-epifysiodesis was considered necessary. However, the progressive swelling of the knee was not explained by the angular knee deformities, since the X-rays showed no signs of osteoarthritis (i.e. no joint space narrowing, osteophyte formation or arthrosis). Ultrasound and contrast-enhanced magnetic resonance imaging (MRI) of the knee were performed. Severe effusion in the knee joint and extensive thickening of the enhancing synovial membrane were observed (see Fig. 2). Since findings on both imaging modalities were highly suspicious for inflammatory pathology, the boy was referred to the pediatric rheumatologist.

The boy presented to the Pediatric Rheumatology Department with progressive swelling in the patellar region of the right knee since 3 months. He reported mild pain during physical activity in the right knee. No morning stiffness, nocturnal pain, fever, history of trauma, tick bite before the onset of the swelling nor previous episodes of joint swelling were reported. The patient did not describe any pain or swelling of other joints. Pre-existing limited range of motion in multiple joints secondary to the GS-related flexion contractures were reported as not progressive. Medical family history was negative for autoimmune disorders. Body height was 111 cm (<− 3 SD) and body weight was 19 kg (+ 0.5 SD; corrected for height). Physical examination revealed warmth and severe swelling of the right knee. No other joints were suspected for arthritis. Laboratory investigations revealed no abnormalities (i.e. differential blood count: normal, leukocyte count: 4.7 × 10^9/L [reference range: 4-14 × 10^9/L], C-reactive protein: 1.1 mg/L [reference range: 0–5 mg/L], antistreptolysin titer: negative, Borrelia serology: negative, human leukocyte antigen-B27: negative, anti-nuclear antibody test [ANA]: negative, IgM rheumatoid factor: 18.0 kU/L [reference range: 0–49 kU/L] and anti-cyclic citrullinated peptide antibody: 9 kAU/L [reference range: 0–25 kAU/L]). The initial differential diagnosis included juvenile idiopathic arthritis (JIA), infectious arthritis, reactive arthritis and malignancy.

Four months after presentation to the pediatric rheumatology service, diagnostic arthroscopy of the right knee was combined with bilateral hemi-epifysiodesis (using 8-plates [orthofix®, Lewisville USA]). During the arthroscopy severe synovitis in the joint space of the right knee was observed. Synovial tissue and fluid were obtained and sent to the Department of Pathology and Medical Microbiology for examination. Synovial fluid assessment revealed high cellularity and mononuclear cell infiltration. Cell analysis showed a significant predominance of macrophages. Additionally, synovial fluid tested negative for any bacterial infection (in particular no detection of *Mycobacterium tuberculosis* or Borrelia spirochetes). Furthermore, the synovial fluid was not suspicious for a malignancy nor crystal-induced arthritis. Microscopic examination of the synovial tissue revealed numerous foamy macrophages with extensive vacuolization, consistent with the previous diagnosis of GS. After the exclusion of infectious arthritis and malignancy, the patient was treated with an intra-articular corticosteroid injection (triamcinolonhexacetonide 1 mg/kg). The swelling reduced significantly during follow-up and had almost disappeared entirely after 6 months.

**Fig. 3:**
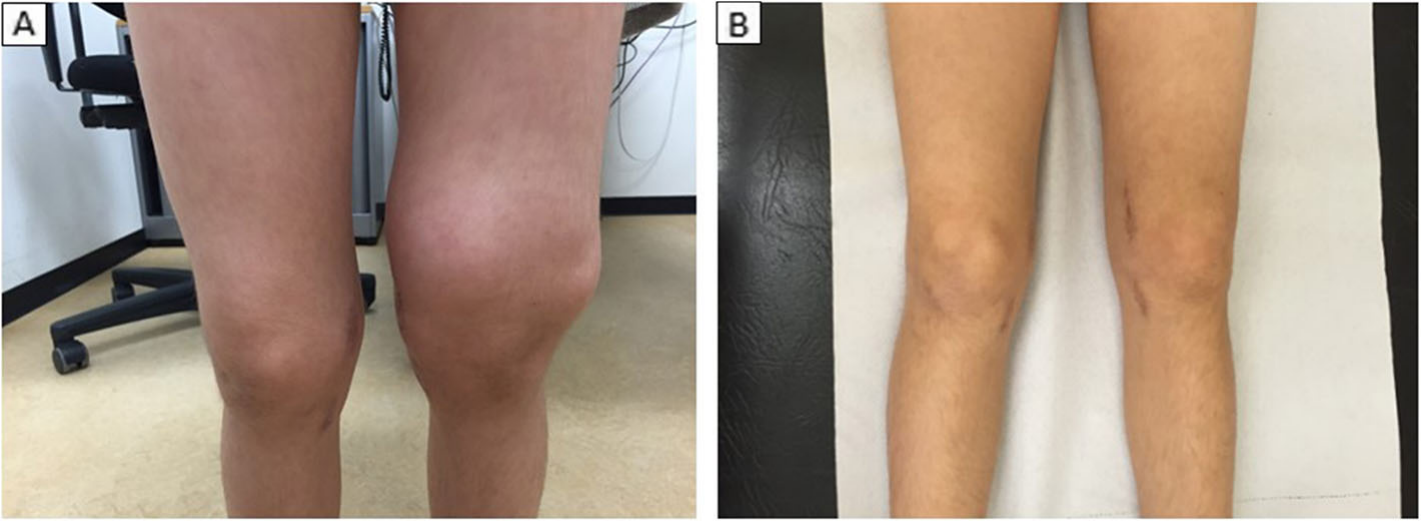
Clinical photographs showing severe swelling of the left knee joint at age 11 (**A**) and the significant improvement 4 months after fluid aspiration and local intra-articular injection of corticosteroid (**B**)

During the next 4 years the patient presented with three more episodes of progressive swelling of the other knee joint. Clinical history, physical examination (except for the location of joint inflammation) and laboratory results were virtually identical to the first episode. Treatment consisted of aspiration of excessive joint fluid and subsequent intra-articular injection of triamcinolonhexacetonide, which was highly effective in all episodes, as displayed in Fig. 3. The duration of clinical remission after injection of corticosteroids was approximately 1 year.

Over the years the patient did not develop any signs suggestive of arthritis in other joints, iridocyclitis, inflammatory bowel disease or skin rashes/psoriasis.

## Discussion and conclusions

GS is a rare LSD which presents with a wide range of clinical features. The musculoskeletal system is one of the organ systems typically affected, resulting in varying degrees of musculoskeletal deformities [1]. A few studies also report patients with articular complaints [5–8], yet knowledge on GS-related joint pathology is limited. Our patient exhibits a remarkable frequency and severity of osteoarticular abnormalities and in addition presented with severe relapsing inflammatory arthritis in both knees, which has never been described before. The differential diagnosis for arthritis in the pediatric population is broad and therefore it can be challenging to obtain a correct diagnosis [9, 10]. In the present case, owing to comprehensive examinations (i.e. physical examination, laboratory tests, radiologic imaging and microscopic examination) multiple possible causes for the recurrent inflammatory joint pathology could be rejected (in particular there were no signs of infectious arthritis, reactive arthritis, osteoarthritis, arthritis secondary to a malignancy or crystal-induced arthritis). However, the clinical results were not conclusive and therefore the primary cause for the recurrent arthritis remains elusive.

A diagnosis which could explain the clinical picture is the JIA subtype: ANA negative oligo-articular JIA. JIA is the most common acquired chronic musculoskeletal pediatric disease and defined by joint inflammation, of unknown origin, that persist for more than 6 weeks and starts before the age of 16 years [10]. JIA is a diagnosis *per exclusionem* [10], and therefore should be considered as a possible diagnosis in this case. However, microscopic examination in our patient showed numerous foamy macrophages with extensive vacuolization in the synovial tissue of the inflamed joint, which is not associated with JIA. Given the evidence of storage products within the macrophages of the inflamed synovial tissue and no conclusive diagnosis, GS itself should be considered as the primary cause for the recurrent arthritis. We hypothesize that the prolonged articular build-up of storage material has induced arthritic cascades, resulting in the development of the inflammatory joint pathology as observed in our patient.

According to the sparse literature on joint complaints in GS, to date, GS cannot be linked directly to joint inflammation. Nevertheless, disease-related arthritis has been described in several other LSDs, such as: Fabry disease (MIM #301500) [11], Farber lipogranulomatosis (MIM #228000) [12–16], Gaucher disease (MIM #230800) [16–19], a-Mannosidosis (MIM #248500) [20], Fucosidosis (MIM #230000) [21], Aspartylglucosaminuria (MIM #208400) [22], Cystinosis (MIM #219800) [23] and in a number of subtypes of Mucopolysaccharidoses (MPSs) [24–26]. Although, some arthritic mechanisms seem to be LSD-specific (e.g. vascular occlusion secondary to bone marrow infiltration with storage cells in Fabry disease [27] and Gaucher disease [28], and crystal-induced arthritis in Cystinosis [29]), systemic as well as local inflammatory processes possibly related to the effects of prolonged articular build-up of storage material in general have been reported. For instance, the abundance of unmetabolized substrates in LSDs might induce aberrant immune responses with subsequent expansion of immune cells of the innate immunity as well as adaptive immunity [26, 30–34]. Moreover, it is thought that storage products in MPSs possibly could act as self-antigens and subsequently might provoke the necessary signals to activate autoimmunity processes [35]. Another potential key player in LSD-related joint inflammation could be the increased activation of Toll-Like Receptor 4 (TLR4), as described in MPSs [26, 36]. TLR4 activation is related to the upregulation of numerous pro-inflammatory proteins, such as chemokines, cytokines (e.g. tumor necrosis factor [TNF] and interleukin-1 [IL1]), nitro oxide products, metalloproteinases, prostanoids and growth factors [26, 36]. Since the cause for storage product deposition in LSDs cannot be eliminated, the trigger for the above described inflammatory cascades is continuously present and therefore eventually might lead to persistent inflammatory activity known as metabolic inflammation [26]. Activation of TLR4 does not only have systemic effects, but also induces local mechanisms which show several similarities to arthritic processes observed in rheumatoid arthritis. For example, the increased levels of TNF and IL1 could result in hyperproliferation of immature synoviocytes and subsequent synovial hyperplasia [26, 36]. Taken into account the rareness of GS [1], future studies using representative GS mouse models (i.e. PPCA −/− mice) [37–39] might be useful to confirm the existence of similar arthritic processes in GS.

In this study, we report, for the first time, the case of a boy affected by GS presenting with relapsing inflammatory arthritis in both knees. Given the evidence of storage material in the inflamed synovial tissue and absence of clues typical for other potential diagnoses, GS itself is considered as the primary cause for the origination of the inflammatory arthritis. Based on the literature about joint inflammation complicating LSDs, we hypothesize that the chronic articular accumulation of GS storage products triggered dysregulation of the innate immune system, resulting in the recurrent episodes of inflammatory joint pathology in our patient. Nevertheless, future identification of other GS patients with joint inflammation is required to corroborate the existence of an arthritic clinical phenotype of GS.

## Data Availability

Data are available from the corresponding author on reasonable request.

## Abbreviations

ANA: Anti-nuclear antibody
GLB1: beta-Galactosidase 1
GS: Galactosialidosis
IL1: Interleukin-1
JIA: Juvenile idiopathic arthritis
LSD: Lysosomal storage disease
NEU1: alpha-N-acetyl Neuraminidase 1
MPS: Mucopolysaccharidosis
MRI: Magnetic resonance imaging
PIP: Proximal interphalangeal
PPCA: Protective Protein/Cathepsin A
TLR4: Toll-Like Receptor 4
TNF: Tumor necrosis factor
